# Intensive exercise program after spinal cord injury (“Full-On”): study protocol for a randomized controlled trial

**DOI:** 10.1186/1745-6215-14-291

**Published:** 2013-09-11

**Authors:** Mary P Galea, Sarah A Dunlop, Glen M Davis, Andrew Nunn, Timothy Geraghty, Ya-seng (Arthur) Hsueh, Leonid Churilov

**Affiliations:** 1 Department of Medicine (Royal Melbourne Hospital), The University of Melbourne, Parkville, VIC 3010, Australia; 2 School of Animal Biology (M317), The University of Western Australia, Crawley, WA 6009, Australia; 3 Discipline of Exercise and Sports Science, The University of Sydney, Lidcombe, NSW 1825, Australia; 4 Victorian Spinal Cord Service, Austin Health, Heidelberg, VIC 3084, Australia; 5 Queensland Spinal Cord Injuries Service, Princess Alexandra Hospital, Woolloongabba, Queensland 4102, Australia; 6 Melbourne School of Population and Global Health, The University of Melbourne, Parkville, VIC 3010, Australia; 7 Florey Institute of Neurosciences and Mental Health, Melbourne Brain Centre (Austin Campus), Heidelberg, VIC 3084, Australia

**Keywords:** Spinal cord injury, Randomized controlled trial, Exercise, Locomotor training, FES

## Abstract

**Background:**

Rehabilitation after spinal cord injury (SCI) has traditionally involved teaching compensatory strategies for identified impairments and deficits in order to improve functional independence. There is some evidence that regular and intensive activity-based therapies, directed at activation of the paralyzed extremities, promotes neurological improvement. The aim of this study is to compare the effects of a 12-week intensive activity-based therapy program for the whole body with a program of upper body exercise.

**Methods/Design:**

A multicenter, parallel group, assessor-blinded randomized controlled trial will be conducted. One hundred eighty-eight participants with spinal cord injury, who have completed their primary rehabilitation at least 6 months prior, will be recruited from five SCI units in Australia and New Zealand. Participants will be randomized to an experimental or control group. Experimental participants will receive a 12-week program of intensive exercise for the whole body, including locomotor training, trunk exercises and functional electrical stimulation-assisted cycling. Control participants will receive a 12-week intensive upper body exercise program. The primary outcome is the American Spinal Injuries Association (ASIA) Motor Score. Secondary outcomes include measurements of sensation, function, pain, psychological measures, quality of life and cost effectiveness. All outcomes will be measured at baseline, 12 weeks, 6 months and 12 months by blinded assessors. Recruitment commenced in January 2011.

**Discussion:**

The results of this trial will determine the effectiveness of a 12-week program of intensive exercise for the whole body in improving neurological recovery after spinal cord injury.

**Trial registration:**

NCT01236976 (10 November 2010), ACTRN12610000498099 (17 June 2010).

## Background

Rehabilitation after spinal cord injury (SCI) has been traditionally based on expectations regarding functional outcomes predicted by the initial level of injury and severity of impairment [1]. It has relied substantially on compensatory strategies for identified impairments and deficits that were considered irremediable, because significant recovery of motor function was not expected beyond that defined by clinical assessments [2]. Thus, in patients with clinically complete injuries, therapy has been primarily directed at activities to improve functional independence, e.g., teaching new strategies to move in bed and transfer into and out of a wheelchair, and provision of assistive devices [2]. This strategy is reliant on strengthening muscles above the spinal lesion level and using leverage, momentum and substitution to move weak or paralyzed parts of the body. While this approach results in improvements of independent function, it does not promote recovery of motor control in the paralyzed limbs [3]. In addition, because of funding limitations available for in-patient rehabilitation, therapists have had to focus on targeting immediate needs for independence in preparation for discharge [2].

Regular physical activity through upper body training is effective in improving cardiorespiratory fitness, muscle strength [4,5] and psychological well-being in the SCI population [6]. However, there is new evidence that regular and intensive *activity-based therapies*, directed at activation of the paralyzed extremities, promotes neurological improvement [2,7-9]. These therapies include partial body-weight-supported locomotor training (LT), functional electrical stimulation (FES)-assisted leg exercise, and exercises to improve control of trunk and lower limb musculature. To date, the interventions have been investigated as single entities; however, Harness et al. [9] conducted a study involving a combination of these interventions. They reported that people with spinal cord injury receiving intensive exercises focused on attempting to regain voluntary motor function below the level of injury showed improvement in motor function, whereas those undertaking a self-directed exercise program did not show any improvements. However, the study had a number of limitations, including a non-randomized design and significant difference between groups in time post-injury.

While most research attention has focused on the benefits of these types of therapy for people with incomplete lesions [10,11], those with complete lesions may also benefit since it is recognized that many people with complete injuries are electrophysiologically incomplete (discomplete) [12,13]. In these cases, patients who are clinically paralyzed show sub-clinical evidence of translesional motor connections [14]. These subclinical responses can take various forms, for example, repeatable responses to reinforcement maneuvers or to strong vibration [15] or the ability to volitionally suppress responses evoked by plantar surface stimulation [16,17]. The objective of this project, therefore, is to determine the effectiveness of an intensive activity-based therapy program for patients with complete and incomplete spinal cord lesions. This will involve LT, FES-assisted leg cycling as well as trunk, upper limb and lower extremity exercises. In the case of complete spinal cord injury, LT will be used to explore the potential for stimulating neurological improvement rather than as a means of attaining functional ambulation [18]. The control intervention will comprise solely upper body exercises.

## Methods

The trial has been registered on ClinicalTrials.gov (NCT01236976) and the Australian and New Zealand Clinical Trials Registry (ACTRN12610000498099).

### Funding

The study is being funded by the Transport Accident Commission (Victorian Neurotrauma Initiative), the Life Time Care and Support Authority (NSW), The University of Melbourne and The University of Western Australia. Further funding for equipment was obtained from the Premier’s Science and Research Fund, South Australia, SpinalCure Australia, and the CatWalk Trust, New Zealand. At the conclusion of the trial, all equipment purchased for the trial will be gifted to the participating hospitals.

### Design

A multicenter, assessor-blinded, randomized controlled trial will be conducted. Experimental group participants will undertake an intensive 12-week program of whole body exercise, including their paralyzed lower limbs, while control group participants will undertake an upper body strength and aerobic fitness training program over the same period. The trial will be conducted in six spinal units in Australia and New Zealand. Ethical approval has been obtained from the Human Research Ethics Committee at each site (see Additional files 1 and 2) and at the University of Melbourne (HREC 1034730). Participants will be provided with information sheets, and written informed consent will be obtained prior to recruitment and baseline assessment.

### Participants

One hundred eighty-eight participants who are at least 6 months post-spinal cord injury will be recruited through referral by medical practitioners or therapists from one of the six participating spinal cord injury units involved or through advertisements placed in newsletters of Spinal Cord Injuries Australia, Independence Australia, on the SCIPA website (http://www.scipa.unimelb.edu.au) and collaborator websites. Those interested in participating will undergo an initial telephone screening survey comprising questions relating to their medical history and the protocol inclusion and exclusion criteria.

### Inclusion criteria

Participants will be included if they:

1. Have sustained a traumatic spinal cord injury a minimum of 6 months prior to consent and have completed their primary rehabilitation

2. Are 18 years or older and able to give informed consent

3. Have a complete or incomplete spinal cord injury between C6 and T12 (as per the International Standards for Neurological Classification of SCI [19]

4. Are able and willing to attend an exercise program three times per week for 12 weeks

5. Are considered by their medical consultant to be fit to undertake the exercise program (documented approval by medical consultant required).

### Exclusion criteria

Participants will not be included if they:

1. Have brachial plexus, cauda equina or peripheral nerve injury

2. Have had recent major trauma or surgery within the last 6 months

3. Have an existing stage 3 or 4 pressure ulcer according to the National Pressure Ulcer Advisory Panel classification [20]

4. Are post-menopausal at the time of injury (females)

5. Have a BMI at injury falling below the lower threshold of the healthy adult reference range

6. Have endocrinopathy or metabolic disorders of the bone, such as Paget’s disease, lytic or renal bone disease, and senile osteoporosis

7. Have a medical history of exposure to medication(s) known to affect mineral or bone metabolism

8. Have chronic systemic diseases, e.g., hepatitis C or HIV-AIDS

9. Have significant impairment or disability, including physical, neurological or psychological impairments, additional to the spinal cord injury

10. Have a history of long bone fracture or family history of fragility fracture

11. Have medical fragility, e.g., a BMI falling below the lower threshold of the healthy adult reference range, or history of recurrent hospital readmissions

12. Have extensive fixed contractures in the upper or lower limbs

13. Have severe spasticity

14. Have uncontrolled neuropathic pain

15. Are likely to experience clinically significant autonomic dysreflexia and/or orthostatic hypotension in response to electrical stimulation or prolonged upright postures

16. Are unable to attend the 6- and 120 month follow-up assessments at their treating spinal unit

17. Have any contraindications to FES such as a cardiac pacemaker, epilepsy, lower limb fracture or pregnancy

18. Have intracranial metal implants

19. Have any other serious medical condition including malignancies, psychiatric, behavioral or drug-dependency problems, which are likely to influence the participant’s ability to cooperate or in the opinion of the study investigator would prevent adherence to the protocol

20. Are participating in any other therapy (including alternative therapies) or taking medications (including herbal preparations) that are not considered to be standard care as per the protocol.

### Randomization

Participants will be randomly assigned to either the experimental or control group with a 1:1 allocation as per a computer-generated randomization schedule stratified by site and injury status [American Spinal Injuries Association Impairment Scale (AIS) A/B or AIS C/D]. The randomization schedule will be under the control of a central randomization unit, independent of the trial, located at Neuroscience Trials Australia. A participant will be considered to have entered the trial once his/her randomization is revealed.

### Intervention

All participants will attend the assigned exercise program for 36 sessions over 12 weeks, with an intended frequency of thrice weekly.

#### Experimental group

Participants in the experimental group will receive a triad of interventions comprising locomotor training, FES-assisted cycling as well as trunk and upper and lower extremity exercises. These interventions will be provided at the participating spinal units.

*Locomotor training* will be provided using a Therastride system (Innoventor, Inc., St Louis, MO, USA). Participants will be assisted to stand on the treadmill in an upright position and suspended in a harness by an overhead pulley at the maximum load necessary to avoid the knees collapsing into flexion. A therapist/assistant will be positioned behind the participant to stabilize the pelvis and trunk, as well as to assist weight shifting and hip rotation during the step cycle. This person will ensure that the trunk and pelvis are not flexed or hyperextended during stepping. Two therapists/assistants will be seated in front and to the side of the participant to provide manual assistance for the lower limbs to facilitate knee extension during stance and knee flexion and toe clearance during swing, maintaining appropriate alignment of the limbs. Manual assistance will be used as needed. Another therapist/assistant will control the treadmill. During the session, the treadmill speed will be adjusted to promote the best stepping pattern at the given body weight load. Speed will be progressively increased as appropriate to a normal walking speed range (0.89-1.34 m•s^-1^). Over the course of the study, the amount of body weight support can be reduced gradually as the participants improve their ability to bear weight on the lower limbs [21].

*FES-assisted cycling* will be provided using a RT300 cycle (Restorative Therapies, Baltimore, MD, USA). Surface electrodes will be applied over the belly of the quadriceps, gluteal and hamstrings muscles according to a standardized protocol. The pedal cadence will be set to 15–50 rev•min^-1^. Stimulation intensity will be gradually increased to a maximum of 140 mA with a pulse width of 0.3 ms at a frequency of 35 Hz [22,23]. Participants will exercise at the maximal power output possible at their level of recovery. They will undertake 30 min of cycling, initially in intermittent periods of exercise with rest breaks, but progressing to longer continuous exercise as deemed appropriate by supervising therapist/assistant. The frequency, power output, interval times and total training duration will be recorded at each training session.

*Trunk and upper and lower limb exercises* will comprise assisted and/or resisted movements aimed at facilitating and strengthening voluntary muscle activity and improving movement quality. These may be provided in lying (e.g., supine, prone, semi-recumbent) and in weight-bearing postures (e.g., kneeling, standing). Task-specific practice of functional tasks, involving moving the upper body over and outside the base of support, will also be undertaken. Participants may also be given exercises in motor imagery, i.e., imagining movements of their body [24]. Project staff will use their clinical judgment to select exercises suitable for each participant and to progress them as appropriate. All exercises selected will be documented in the participant source notes.

#### Control participants

Participants in the control group will receive an upper body strength and fitness program provided three times per week for 12 weeks. This program may be provided at the spinal unit or an appropriately equipped gymnasium as approved by the Project Committee. The program will be a circuit-based exercise program incorporating resistance and cardiorespiratory training. For this protocol, the circuit will comprise several stations using different pieces of equipment and with minimal time to rotate between stations. The participants will be supervised by a therapist and/or clinical exercise instructor, and exercises will be progressed as appropriate to build strength and endurance in the intensity range of 6–20 repetition maximum (RM). Exercises could include hand cycling, chest press, boxing, lateral pull-downs and rowing. Guidelines for the content and delivery of exercises will be clearly outlined in a handbook to ensure standardization across sites.

#### Wash-out and follow-up period

For 3 weeks prior to the intervention, participants will cease any formal intensive exercise programs in which they are participating. During the program, participants will not undertake any formal intensive exercise programs in addition to those provided in the study protocol. At the end of the 12-week intervention period, there will be no restriction placed on participants with respect to undertaking further exercise.

#### Quality assurance

To ensure that treatments are of a high standard and delivered in accordance with the trial protocol, therapists responsible for the administration of the exercise programs will attend workshops where they will be trained in the delivery of the treatment program and in assessment procedures. The workshops will cover locomotor training (4 days), FES-assisted cycling training (2 days), trunk training (2 days), upper body training (1 day) and assessment procedures (1 day). Therapists will also be provided with a written protocol, and standardized case report forms for documentation of assessments and interventions provided.

### Outcome assessments

Assessments will occur at baseline, 12 weeks after the commencement of the intervention, and then at 6 months and 12 months after randomization. All assessments will be undertaken by therapists blinded to group allocation. Any inadvertent unblinding of assessors will be reported. In addition, the success of blinding will be estimated by asking assessors to guess the participant’s group allocation at the completion of each post-randomization assessment.

### Primary outcome

#### The primary outcome measure is the American Spinal Injuries Association (ASIA) motor score at 12 weeks

The ASIA Motor Score is derived from part of the assessment for the International Standards for Neurological Classification of Spinal Cord Injury [19]. It involves testing the strength of ten key muscles on each side of the body in the supine position (e.g., elbow flexors, wrist extensors, hip flexors, quadriceps, dorsiflexors) on a scale of 0 = no contraction to 5 = normal resistance through full range of motion. Scores are summed to give a total possible score of 50 for the upper extremities and 50 for the lower extremities.

### Secondary outcome measures are

#### ASIA motor and sensory scores at 6 months and 12 months

The ASIA Motor Assessment will be undertaken as described above. The ASIA Sensory assessment is also part of the assessment for the International Standard for Neurological Classification of Spinal Cord Injury. It involves testing pinprick and light touch sensation at key points representing each dermatome. Pin-prick and light-touch sensation of each dermatome is separately scored on a 3-point scale (0, 1 and 2). Scores will be summed to give a total possible score of 224 where a higher score indicates better sensation than a lower score.

#### Leg exercise capacity test at 12 weeks, 6 months and 12 months

Participants will perform a graded exercise test using their legs (FES cycling), following the protocol of Heesterbeek et al. [25]. The electrical stimulation current amplitude will be increased manually from the lowest current amplitude that evokes minimum muscle contraction up to 100% amplitude of the cycle’s stimulator output (~140 mA). The timing of FES stimulation current amplitude increments will be based on the prevailing heart rate response between resting heart and age-predicted maximum heart rate. The measures of interest will be change of peak power output (W), peak heart rate (b•min^-1^) and change of sub-maximal heart rate (b•min^-1^) during the 20 W and 40 W stages and during the incremental portion of the leg cycling.

#### Spinal cord independence measure (SCIM) at 12 weeks, 6 months and 12 months

The SCIM was designed specifically for patients with spinal cord injuries. It focuses on the spinal cord injured person’s ability to perform basic everyday tasks and takes into consideration the economic burden of disability as well as the impact of disability on the participant’s overall medical condition and comfort [26,27]. The SCIM consists of three complementary subscales: ‘Self care,’ ‘Respiration and sphincter management’ and ‘Mobility’.

#### Anthropometry at 12 weeks, 6 months and 12 months

Body mass index (BMI) will be estimated from body mass and lying stature. Skin fold calipers will be used to assess change in body adiposity at 12 sites on the body while the participant is lying horizontal [28]. Abdominal circumference, chest circumference and leg circumferences will be assessed at the greatest girth-points.

#### Measures of trunk function at 12 weeks, 6 months and 12 months

*Maximal balance range*[29] will be measured by asking participants to reach as far forward as they can without falling and then return to their starting position. The distance reached will be measured using a sway meter, a 40-cm hinged rod fastened by a firm belt to the participants’ chest at the level of the axilla and extending in a horizontal plane from the body. A ballpoint pen mounted at the end of the rod will record the movements of the upper body on a sheet of graph paper fixed to the top of a height-adjustable table with a meter-long ruler taped to the side. The resultant trace will be measured in terms of the maximal anteroposterior (AP) displacement in centimeters traversed by the pen. The participants will have two attempts at the test, with the longer distance (maximal AP distance moved) taken as the test result. A long distance is considered a better performance. This score will be corrected for body height (score × mean height/participants’ height) measured from the center of the sway meter strap to the top of the seat.

*The Spinal Cord Injury-Falls Concern Scale (SCI-FCS)*[30] is a questionnaire adapted from the Falls Efficacy Scale-International [31] and worded appropriately for activities undertaken by people in wheelchairs. Participants will be asked to record on a 4-point scale how concerned they are about falling when performing each activity. A score of 1 reflects ‘not at all concerned’ and a score of 4 reflects ‘very concerned.’ Scores on each item will be summed.

#### Spasticity at 12 weeks, 6 months and 12 months

Spasticity over the previous week will be measured via self-report using the Penn Spasm Frequency Score (PSFS) [32,33]:

0 = No spasms

1 = Spasms induced by stimulation

2 = Spasms occurring less than once per hour

3 = Spasms occurring more than once per hour

4 = Spasms occurring more than 10 times per hour

Any change in the dosage of anti-spasticity medication during the intervention period will also be monitored.

#### Multidimensional pain inventory (spinal cord injury version) at 12 weeks, 6 months and 12 months

Pain will be measured using the spinal cord injury version of the Multidimensional Pain Inventory (SCI-MPI) [34,35]. This is a comprehensive instrument designed to assess a range of self-reported behavioral and psychosocial factors associated with chronic pain syndromes and has been validated in the spinal cord injury population.

#### Walking tests at 12 weeks, 6 months and 12 months

Walking tests will be conducted only in participants capable of ambulation. The 6-Minute Walk Test (6MWT) is a self-paced task that measures the distance that a participant can walk in 6 min. The participant may stop to rest at any point. The test may be performed either indoors or outdoors, along a long, flat, straight and hard surface, preferably 30 m in length, marked every 3 m, with a turnaround point marked with a cone. The distance (total distance walked rounded to the nearest meter) will be measured. The fatigue level will also be reported.

The 10 Meter Walk Test (10MWT) measures the time required to walk 10 m. Participants will walk at their preferred walking speed. They may use an assistive device and must wear shoes. To minimize the effects of acceleration and deceleration, participants will commence walking at least 2 m before the start of the 10 m walking track and continue for 2 m beyond the end of the track (14 m altogether). The time (seconds) will be measured over the intermediate 10 m and walking speed calculated in m•s^-1^.

#### Psychological measures at 12 weeks, 6 months and 12 months

Stress will be measured using the Perceived Stress Scale (PSS) [36], which is the most widely used psychological instrument for measuring the perception of stress. Depression will be measured using the Hospital Anxiety and Depression Scale (HADS) [37]. Physical Self-Concept will be measured using the Multidimensional Health Locus of Control [38]. Self-Efficacy will be measured using the Moorong Self-Efficacy Scale [39,40] and Self Esteem using the Rosenberg Self-Esteem Scale [41]. All of these tools have previously been used in people with spinal cord injury.

#### Quality of life at 12 weeks, 6 months and 12 months

The Health Utilities Index Mark 3 (HUI3) is a measure of health-related quality of life widely used around the world in population health surveys, clinical studies and cost-utility analyses. HUI3 includes eight attributes (vision, hearing, speech, ambulation, dexterity, emotion, cognition and pain), with five or six levels for each attribute. The HUI3 is able to discriminate various aspects of burden associated with chronic conditions as well as describing the differences in overall health-related quality of life levels [42]. In addition, the Assessment of Quality of Life (AQoL)-8, which is a self-administered 8-item questionnaire [43], is also used for two reasons. One is that this instrument was developed relatively more recently based on the utility value of Australian population. The other is to provide an alternative source of information from a different instrument.

The WHOQoL-Bref questionnaire was designed by an international collaboration on quality of life by the World Health Organization. It contains 26 questions about many different aspects of quality of life, with some additional questions about the person and his/her health. It has been found to be a valid measure in people with spinal cord injury [44].

### Economic outcomes

Economic evaluation will determine whether the experimental intervention is cost-effective compared to the control intervention. The incremental cost-effectiveness analysis will measure incremental costs in the two groups in relation to the AIS Motor score, divided by the incremental change in health utility and quality of life measures. This study employs a societal perspective. Therefore, all relevant costs associated with participants and delivery of experimental and control interventions will be included. The cost of treatment will be estimated using standard costs for therapy and actual costs of training equipment expressed as a mean cost of treatment per participant. Community-based resource use from the time of randomization will include data on visits to GPs, specialists or other health care providers, pharmaceutical costs, as well as data on resource use specifically relating to levels of independent functioning (aids, equipment, community services, e.g., home help, home maintenance, meals on wheels, transport, formal and informal care, cost of home modifications, etc.). The potential loss of productivity of the participant will also be measured.

Treatment effectiveness will be determined by the primary endpoint, as well as the health utility and quality of life measures. Cost-effectiveness will be calculated as the cost per patient treated. These calculations will be based on the number of participants effectively treated in the intervention group minus the number of participants who also meet the criteria of ‘effectively treated’ in the control group. Lifetime cost and effectiveness will be modeled using decision-tree and Markov processes.

### Statistical analyses

#### Sample size

This power analysis is for a one-way fixed effects analysis of covariance with two levels that correspond to experimental and control arms of the study. This analysis includes baseline value of AIS Motor Score as a covariate and uses week 12 values as the primary outcome. Available pilot data [9] demonstrate a mean change in the AIS Motor Score of 4.8 (SD = 1.0) in an intensive exercise group compared to a mean change of 0.1 (SD = 0.5) points in the control group from the comparable baseline of 40. Assuming the more conservative difference in mean changes in AIS Motor Score between the groups of 4.0, a similar baseline mean AIS Motor score of 40 and a correlation between the baseline and week 12 ASIA Motor score of 0.8, the resulting analysis of covariance effect size (f) is 0.22. The corresponding total sample size of 150 participants equally distributed between experimental and control arms will yield power of 0.8 to reject the null hypothesis at a 0.05 level of significance. The analysis of variance is non-directional (i.e., two-tailed), which means that an effect in either direction will be interpreted. Allowing for 20% dropout rate, the final sample size for this study is calculated to be 188 patients equally distributed between experimental and control arms.

#### Statistical analysis

The primary and secondary endpoints analyses will be conducted by an independent statistician on an intention-to treat basis and using the full data set comprising all randomized participants. In addition, a *per protocol* analysis of the primary outcome will be reported. The data set for this analysis will comprise participants who adhered to all aspects of the protocol and received at least 80% of the training sessions. Multiple imputation of missing values will be conducted. All variables will be expressed as means, SD or median (IQR), unless otherwise indicated. Baseline comparisons between the two arms of the study will be made using means or medians and proportions with 95% confidence intervals as appropriate.

All endpoints and analyses have been prospectively categorized as either primary or secondary. Differences in both primary and secondary endpoints between the two arms of the study will be tested independently at the 0.05 level of significance, using a one-way ANCOVA model that will include the baseline value of the given outcome in question as a covariate. No formal adjustments will be undertaken to constrain the overall type I error associated with the secondary analyses. Their purpose is to supplement evidence from the confirmatory primary analysis to help more fully characterize the treatment effect. Results from the secondary analyses will be interpreted in this context.

#### Data integrity and management

Data will be stored electronically on a database with secured and restricted access. Data transfer will be encrypted and any information capable of identifying individuals removed.

#### Withdrawals

A participant will be considered to have withdrawn from the trial when consent is revoked or if the participant cannot be contacted or located. If this occurs, no further assessments will be performed. Participants will not be withdrawn from the trial for protocol violations.

#### Monitoring

The trial will be overseen and monitored by a Program Manager. The Program Manager will visit each site to examine trial procedures, ensure data quality and monitor compliance with the trial protocol. All adverse events and serious adverse events will be recorded according to standard procedures. Three safety variables will be monitored and documented throughout the trial. These are self-reported pain (using an 11-point category rating scale), blood pressure and skin irritation from the stimulating electrodes or body weight support harness. However, only two safety variables (pain and blood pressure) are considered serious enough to warrant inclusion in two unblinded safety interim analyses. These analyses will be undertaken when 60 and then 120 participants have completed the post-intervention assessment. It will be done by an Independent Data Safety Monitoring Board comprising a statistician and two rehabilitation doctors. If there are concerns about the safety of participants, this board will make a recommendation to the trial steering committee about continuing, stopping or modifying the trial. The Haybittle-Peto procedure for generating early stopping boundaries will be used for pain and blood pressure. A recommendation of early termination for safety reasons because of pain (mean margin of 4/10) or blood pressure (mean margin of 40 mmHg) will be considered by the Independent Data Safety Monitoring Board if the corresponding Haybittle-Peto boundary (*p* = 0.003, Z = 3) at a given interim analysis is crossed. No formal interim analyses for efficacy or futility are planned.

## Discussion

This trial will provide information about the effectiveness of an intensive full-body exercise program in promoting neurological improvement. This is important for understanding the therapeutic effect of rehabilitation independent of drug or cellular interventions, since rehabilitation may confound the outcome of these interventions [45]. The mechanisms by which exercise programs improve function after SCI also need to be better understood because they allow for development of new and more effective therapeutic strategies. In this study, the control intervention is not standard care but an active intervention, involving an intensive exercise program for the upper body. This ensures that the intensity and group dynamics of the both exercise programs are similar, so that the effects of adding exercise of the paralyzed lower limbs can be identified.

This trial will adhere to key methodological principles important for minimizing bias (International Conference on Harmonisation Good Clinical Practice annotated by the Therapeutic Goods Administration of Australia) [46] and will be reported according to the CONSORT guidelines [47]. For example, allocation will be concealed and randomized, assessors will be blinded, and analyses will be performed on an intention-to-treat basis. Therapists and participants will not be blinded because of the nature of the intervention. One primary outcome and a number of secondary outcomes will be used. The primary outcome reflects neurological function. The secondary outcomes include measures of impairment, activity limitation and participation restriction, and encompass both objective measures as well as participants’ perceptions. Importantly, all adverse events will be rigorously documented so that the safety of the interventions can be evaluated. It is anticipated that this trial will take 3 years to complete.

## Trial status

Recruitment commenced in January 2011, with the first participant randomized in February 2011. Recruitment will continue until the end of 2013. The 12-month follow-up assessments will be completed in 2014.

## Competing interests

There are no competing interests.

## Authors’ contributions

MPG, SAD, GMD, AN and TG were responsible for the design of the trial and secured funding. MPG is responsible for the coordination and management of the trial. Y-SH is responsible for the cost-effectiveness analyses and LC is responsible for statistical design and analysis. All authors have read and approved the final manuscript.

## Supplementary Material

Additional file 1: Figure S1 Schedule of enrollment, interventions and assessments. Click here for file

Additional file 2 Names of Human Research Ethics Committees providing approval for the study. Click here for file
