# Supplementary material for: Intensive exercise program after spinal cord injury (“Full-On”): study protocol for a randomized controlled trial
Source: Trials. 2013 Sep 11;14:291. doi: 10.1186/1745-6215-14-291 (PMC3848453; doi:10.1186/1745-6215-14-291)
Supplement: Additional file 1: Figure S1 — Schedule of enrollment, interventions and assessments. [file 1745-6215-14-291-S1.doc]

Additional file 1: Figure S1. Schedule of enrolment, interventions, and assessments

|  | **Enrolment** | **Allocation** | **Intervention** | **Post-intervention** | | |
| --- | --- | --- | --- | --- | --- | --- |
| **TIMEPOINT**** | ***-t_1_*** | **0** | **12 weeks** | ***12 wks*** | ***6 mths*** | ***12 mths*** |
| **ENROLMENT:** |  |  |  |  |  |  |
| **Eligibility screen** | X |  |  |  |  |  |
| **Informed consent** | X |  |  |  |  |  |
| **Medical clearance** | X |  |  |  |  |  |
| **Allocation** |  | X |  |  |  |  |
| **INTERVENTIONS:** |  |  |  |  |  |  |
| ***Whole Body Exercise*** |  |  |  |  |  |  |
| ***Upper Body Exercise*** |  |  |  |  |  |  |
| **ASSESSMENTS*:** |  |  |  |  |  |  |
| ***ASIA Motor Score***  ***(1^o^ outcome measure)*** | X |  |  | X | X | X |
| ***ASIA Sensory Score*** | X |  |  | X | X | X |
| ***Leg Exercise Capacity Test*** | X |  |  | X | X | X |
| ***SCIM*** | X |  |  | X | X | X |
| ***Anthropometry*** | X |  |  | X | X | X |
| ***Measures of trunk function*** | X |  |  | X | X | X |
| ***Spasticity*** | X |  |  | X | X | X |
| ***MPI (SCI version)*** | X |  |  | X | X | X |
| ***Walking tests (if applicable)*** | X |  |  | X | X | X |
| ***Psychological measures (PSS, HADS, MHLC, Self-Efficacy, Self Esteem)*** | X |  |  | X | X | X |
| ***Quality of Life***  ***(HUI3 and AQoL)*** | X |  |  | X | X | X |
| ***Community-based resource use (monthly)*** | X |  | X | X | X | X |

*1-week window allowed for assessments

SCIM=Spinal Cord Independence Measure; MPI (SCI version)=Multidimensional Pain Inventory (Spinal Cord Injury version); PSS-Perceived Stress Scale, HADS=Hospital Anxiety and Depression Scale; MHLC=Multidimensional Health Locus of Control; HUI3=Health Utilities Index Mark 3; AQoL=Assessment of Quality of Life
