# Supplementary material for: Intensive exercise program after spinal cord injury (“Full-On”): study protocol for a randomized controlled trial
Source: Trials. 2013 Sep 11;14:291. doi: 10.1186/1745-6215-14-291 (PMC3848453; doi:10.1186/1745-6215-14-291)
Supplement: Additional file 2 — Names of Human Research Ethics Committees providing approval for the study. [file 1745-6215-14-291-S2.docx]

**Intensive Exercise Program after Spinal Cord Injury (“Full-On”): study protocol for a randomised controlled trial**

Human Research Ethics Committees which provided approval for this study are listed below:

The University of Melbourne Human Research Ethics Committee (Trial Sponsor)

Lead Ethics Committee for the two sites in New South Wales – Northern Sydney Local Health District Human Research Ethics Committee

- Research Governance for the Royal Rehabilitation Centre site – Royal Rehabilitation Centre Research Governance Office
- Research Governance for the Prince of Wales Hospital site – South Eastern Sydney Local Health District Research Governance Office

Victoria (Austin Health site) – Austin Health Human Research Ethics Committee

Western Australia (Royal Perth Hospital site) – Royal Perth Hospital Human Research Ethics Committee

South Australia (Hampstead Rehabilitation Centre site) – Royal Adelaide Hospital Research Ethics Committee

New Zealand – Multi-region Ethics Committee (Ministry of Health)
